# Validity of the *iLOAD*® app for resistance training monitoring

**DOI:** 10.7717/peerj.7372

**Published:** 2019-08-07

**Authors:** Evandro Claudino de Sá, André Ricarte Medeiros, André Santana Ferreira, Amador García Ramos, Danica Janicijevic, Daniel Boullosa

**Affiliations:** 1 iLOAD Solutions, Brasilia, Brazil; 2 Catholic University of Brasilia, Águas Claras, Brazil; 3 Department of Sports Sciences and Physical Conditioning, Faculty of Education, Universidad Católica de la Santísima Concepción, Concepción, Chile; 4 Department of Physical Education and Sport, Faculty of Sport Sciences, University of Granada, Granada, Spain; 5 Faculty of sport and physical education, The Research Centre, University of Belgrade, Belgrade, Serbia; 6 College of Healthcare Sciences, James Cook University, Townsville, Australia; 7 Faculty of Healthcare Sciences, University of Brasilia, Brasilia, Brazil

**Keywords:** Smartphone application, Squat, Total work, Velocity-based training

## Abstract

**Background:**

This study aimed (I) to assess the inter-rater agreement for measuring the mean velocity (MV) of the barbell with the *iLOAD*® app, and (II) to compare the magnitude of the MV and total work of a training session between the *iLOAD*® app and a linear encoder (reference method).

**Method:**

Sixteen young healthy individuals (four women and 12 men) were tested in two sessions separated by 48 h. The 10 repetition maximum (RM) load was determined in the first testing session in the half squat exercise. The second testing session consisted of 3 sets of 10 repetitions during the half squat exercise performed against the 10RM load. Both the *iLOAD*® app and a linear encoder were used to calculate the MV and total work of each training set. MV was recorded with the *iLOAD*® app by two independent researchers to evaluate the inter-rater agreement.

**Results:**

Trivial differences and nearly perfect correlations were observed between raters for the MV values collected under individual sets (effect size [ES] ≤ 0.02, *r* ≥ 0.987), as well as for the whole training session (*ES* = 0.01, *r* = 0.997). Trivial-small differences and nearly perfect correlations were observed between the iLOAD® app and the linear encoder (*Chronojump*, Barcelona, Spain) for MV (*EV* ≤ 0.25, *r* ≥ 0.903) and total work (*ES* ≤ 0.05, *r* ≥ 0.973). Bland-Altman plots did not reveal heteroscedasticity of the errors between the *iLOAD*® app and the linear encoder for MV (*r*^2^ = 0.010) and total work (*r*^2^ < 0.001).

**Conclusions:**

*iLOAD*® is a valid smartphone app which can provide real-time feedback of the MV and total work completed in a set of multiple repetitions in the half squat exercise.

## Introduction

Resistance training (RT) is a fundamental part of training for competitive athletes (Suchomel, Nimphius & Stone, 2016) as well as for the general population (Myers, Beam & Fakhoury, 2017; Guizelini et al., 2018). RT does not only provide an essential stimulus for the development of muscle mass and strength (Schoenfeld, 2013; Suchomel et al., 2018), but it may also lead to a better performance in different tasks such as jumping, running, sprinting, kicking, and shooting (Folland & Williams, 2007; Suchomel, Nimphius & Stone, 2016). The monitoring of RT is important for the management of fatigue and to explore the association between the RT performed and the chronic adaptations induced in physical performance (Scott et al., 2016; Fernandes, Lamb & Twist, 2018). A wide range of tools are currently available for RT monitoring, including perceived exertion scales (Singh et al., 2007; Robertson et al., 2008), linear position transducers (Harris et al., 2010), force plates (Dugan et al., 2004), contact mats (Crewther et al., 2011), high-speed cameras (Sañudo et al., 2016), isokinetic dynamometers (Ratamess et al., 2016) or accelerometers (Balsalobre-Fernández et al., 2016). These tools are frequently used to evaluate the effect of RT programs.

One of the tools that has received more scientific attention in recent years for physical activity and RT monitoring are smartphone applications (i.e., apps) (Peart, Balsalobre-Fernandez & Shaw, 2018). Smartphone apps are popular due to their low cost and high portability. These apps collect data using different technologies, such as global positioning systems, accelerometers, gyroscopes, microphones, or high-speed cameras (Higgins, 2016; Peart, Balsalobre-Fernandez & Shaw, 2018). It is currently accepted that movement velocity is one of the most important variables for monitoring and prescribing RT programs (González-Badillo, Marques & Sánchez-Medina, 2011; Jovanovic & Flanagan, 2014). Although linear position transducers and inertial measurement units are the two most commonly used devices for monitoring movement velocity during RT, smartphone apps are beginning to be used for this purpose (Perez-Castilla et al., 2019). For example, the *PowerLift*^®^ app has been validated for measuring mean velocity (MV) of individual repetitions during several RT exercises (Balsalobre-Fernández et al., 2017; Perez-Castilla et al., 2019). However, a limitation of the *PowerLift^®^* app is that it does not provide real-time velocity feedback because the user should manually select the start and end point of each repetition. Moreover, the current version of the *PowerLift^®^* does not provide the average velocity of a set of multiple repetitions. Therefore, it would be necessary to develop a smartphone App that provides real-time feedback of the average velocity of a set of multiple repetitions. In addition, to the best of our knowledge, there are no apps providing the total work during resistance exercises. This information would be of great interest as work has been suggested to be an appropriate parameter for quantification of training volume in different RT protocols (McBride et al., 2009).

To address this gap, our research group has recently developed the *iLOAD*^®^ app. The *iLOAD^®^* app provides the MV (m s^−1^) and total work (J) of a training set in real-time using the smartphone’s timer and calculator. However, the *iLOAD*^®^ app has not been scientifically validated. Thus, the main objective of this study was to validate the *iLOAD^®^* app for RT monitoring during the half squat exercise. The half squat exercise was chosen because it is related to daily physical activities such as standing up from a sitting position and it has also been demonstrated to be effective for strength and muscle mass development, performance enhancement and injury prevention (Schoenfeld, 2010; Hartmann, Wirth & Klusemann, 2013). Specifically, in this study we aimed: (I) to assess the inter-rater agreement for measuring the MV with the *iLOAD^®^* app; and (II) to compare the magnitude of the MV and total work of a training session between the *iLOAD^®^* app and a linear encoder (reference method). It was hypothesized that a high level of agreement would be obtained between raters (rater 1 vs. rater 2) and devices (*iLOAD^®^* app vs. linear encoder). Of note, it would be important to obtain a high level of agreement between raters to confirm that the outcomes collected with the *iLOAD^®^* app do not depend on the rater. The confirmation of our hypotheses would place the *iLOAD^®^* app as a cheap, portable and time-efficient tool for RT monitoring.

## Materials & Methods

### Participants

Sixteen young healthy individuals, four women (mean ± standard deviation [SD]; age = 29.5 ± 7.2 years, body height = 1.61 ± 0.07 m, body mass = 58.7 ± 6.1 kg, squat one repetition maximum [1RM] = 53.0 ± 18.8 kg) and 12 men (mean ± SD; age = 27.4 ± 7.2 years, body height = 1.76 ± 0.05 m, body mass = 78.7 ± 8.2 kg, squat 1RM = 102.7 ± 15.4 kg), volunteered to participate in this study. All participants were familiar with the half squat exercise and had at least one year of RT experience. Prior to testing and after detailed explanation of the procedures and risks of the study, participants gave their written consent to participate in the study. The study protocol adhered to the tenets of the Declaration of Helsinki and was approved by the Catholic University of Brasilia (54813016.0.0000.0029).

### Study design

**Figure 1:**
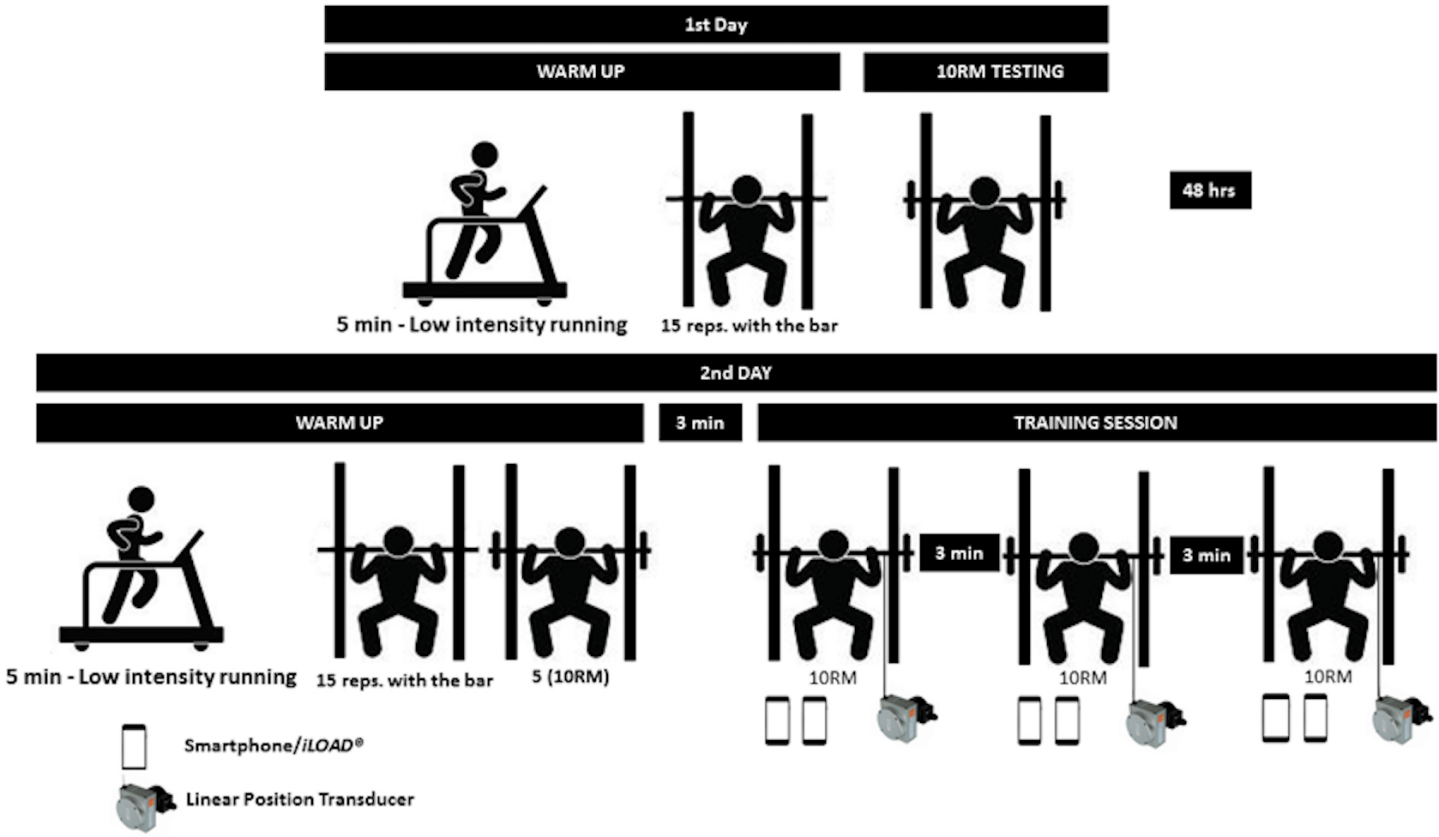
Overview of the experimental design.

This study was designed to determine the validity of the *iLOAD^®^* app for monitoring MV and total work during a RT session (Fig. 1). Participants came to the laboratory on two occasions separated by 48 h. The 10RM load was determined in the first testing session during the half squat exercise. In the second testing session participants were instructed to perform three sets of 10 repetitions during the half squat exercise against the 10RM load. Participants completed 9.9 ± 0.5, 9.5 ± 1.3, and 9.7 ± 1.3 repetitions during the first, second and third set, respectively. Both the *iLOAD^®^* app (v 1.0; ILoad Solutions, Brasilia, Brazil) and a linear encoder (*Chronojump*, Barcelona, Spain) were used to calculate MV and total work of each training set. After familiarization with the *iLOAD^®^* app, two independent researchers recorded MV over the training sets with the *iLOAD^®^* app to evaluate the inter-rater agreement. The average value of both raters was considered to explore the concurrent validity of the *iLOAD^®^* app with respect to the linear encoder. Both testing sessions were performed at the same time of the day for each participant.

### Procedures

All measurements were conducted at the same laboratory (“Laboratório de Estudos da Força” of “Universidade Católica de Brasília”). Anthropometric data was assessed at the beginning of the first session with a stadiometer (ES2040; Sanny, São Paulo, Brazil) and an electronic scale (W110 H LED; Welmy, Santa Bárbara d’Oeste, Brazil). The vertical distance of the barbell for each participant between a knee angle of 90° and the standing position (hips and knees fully extended and feet flat on the floor) was determined with a measuring tape (E095; Stamaco, São Paulo, Brazil). This distance was introduced in the *iLOAD^®^* app for the computations of MV and total work. This measurement was collected while the participants held the unloaded Smith machine barbell (17 kg). The 90° knee angle was determined with a manual goniometer (187-907; MITUTOYO^®^, São Paulo, Brazil). To ensure a consistent countermovement depth during all repetitions, an elastic cord was positioned to contact with the participants’ buttocks when they reached a 90° knee angle. In addition, an adhesive tape was located on the ground to instruct the participants to place their feet always in the same position. After these measurements, participants completed a warm-up consisting of 5 min of submaximal running on a treadmill at a self-selected pace, followed by 15 repetitions with the 17 kg bar of the Smith machine used in the present study (Power Tech, Righetto, São Paulo, Brazil). Thereafter, the 10RM load during the half squat exercise was evaluated following the protocol proposed by the *American College of Sports Medicine* (i.e., four attempts separated with a 3-min recovery interval) (Pescatello et al., 2014). The initial load corresponded to 70% of the self-perceived 10RM, and it was progressively increased until the participant could not complete more than 10 repetitions.

The warm-up of the second testing session consisted of 5 min of submaximal running on a treadmill at a self-selected pace, 15 repetitions with the unloaded Smith machine barbell, and five repetitions with the previously determined 10RM load. Three minutes after completing the last warm-up set, participants were instructed to perform three sets of 10 repetitions during the half squat exercise against the 10RM load with 3 min of rest between sets. Participants were instructed to complete all sets as quickly as possible maintaining the same range of motion during all repetitions. All sets started with a ’go’ instruction from one of the raters, and were considered finished when the concentric phase of the last repetition was completed.

### Data acquisition and analysis

Mean velocity (MV) and total work of the three sets were calculated from the recordings of both the *iLOAD^®^* app and the linear encoder:

- *iLOAD^®^ app*: The *iLOAD^®^* app was installed in two smartphones (5S, iPhone, USA) with an actualized operating system (11.2, IOS, USA). Two independent raters were positioned in front of the participants and recorded the time needed to complete each set. The raters were familiarized with procedures during two preliminary sessions which consisted of the same protocols. The inputs of the *iLOAD^®^* app for each set were the load (in kilograms), number of repetitions, vertical distance of the barbell, and time needed to complete the set (in seconds). The time needed to complete each set was determined by the chronometer of the smartphone. The smartphone’s chronometer was initiated after the ’go’ signal used to indicate the start of the set in the *iLOAD^®^* app and it was stopped when the subject completed the last repetition of the set (i.e., when the hips and knees reached full extension). Of note, because of the greater mechanochemical efficiency during the eccentric action (i.e., negative work) when compared to the concentric action (i.e., positive work) of a single complete repetition (De Looze et al., 1994; Ryschon et al., 1997), a factor of 1.33 rather than 2 was considered for the sum of the concentric and eccentric phases for total work calculations (Bloomer et al., 2006). In addition, the iLOAD^®^ app was used in the ‘squatting’ mode that considers the user’s body mass with a weighting factor of 0.88 (Bloomer et al., 2006) for computing total work. The MV and total work of each set were automatically calculated by the *iLOAD^®^* app as follows:

(1) \begin{eqnarray*}Mean~velocity(m/s)= \frac{Number~of~repetitions\times 2\times Vertical~distance~(m)}{Time~needed~to~complete~the~set~(s)} \end{eqnarray*}

(2) \begin{eqnarray*}Total~work \left( J \right) =1.33\times Vertical~distance \left( m \right) \times Number~of~repetitions\nonumber\\\displaystyle \times \left[ \left( Body~mass\times 0.88 \right) +Load \left( kg \right) \right] \times 9.81 \left( m/{s}^{2} \right) \end{eqnarray*}

- *Linear encoder*: The linear encoder (*Chronojump*, Barcelona, Spain) was connected to a laptop (NP540U3C, Samsung Electronics Co., China) with custom made software (1.8.1-95, Chronojump, Barcelona, Spain). The cable of the linear encoder was fixed perpendicularly to the barbell and recorded displacement-time data at 1,000 Hz. An Excel^®^ spreadsheet was used to calculate the MV and total work from the raw displacement-time data provided by the software as follows:

(3) \begin{eqnarray*}Mean~velocity~(m/s)= \frac{Downward~displacement \left( m \right) +Upward~displacement~(m)}{Time~needed~to~complete~the~set~(s)} \end{eqnarray*}

(4) \begin{eqnarray*}Total~work \left( J \right) =1.33\times Vertical~distance \left( m \right) \times Number~of~repetitions\nonumber\\\displaystyle \times \left[ \left( Body~mass\times 0.88 \right) +Load \left( kg \right) \right] \times 9.81 \left( m/{s}^{2} \right) \end{eqnarray*}

### Statistical analysis

Descriptive data are presented as means and SD. Normality of the distribution was confirmed by the Shapiro–Wilk test (*p* > 0.05). The inter-rater agreement for the recordings of MV with the *iLOAD^®^* app, as well as the concurrent validity of the *iLOAD^®^* app respect to the linear encoder for measuring MV and total work were assessed by independent samples t-tests, Cohen’s *d* effect size (ES and 95% confidence interval [CI]), Pearson’s correlation coefficients (*r*) and Bland-Altman plots. Note that the total work was not compared between raters because its value does not depend on the time recorded by the raters (see Eq. (2)). The scales proposed by Hopkins et al. (2009) were used to interpret the magnitude of the ES (trivial <0.20, small = 0.20–0.60, moderate >0.60–1.20, large >1.20–2.00, and extremely large >2.00) and *r* coefficients (trivial <0.10, small = 0.10–0.30, moderate >0.30–0.50, high >0.50–0.70, very high >0.70–0.90, or practically perfect >0.90). Heteroscedasticity of error was defined as a *r*^2^ > 0.10 (Atkinson & Nevill, 1998). All statistical analyses were performed using SPSS software version 22.0 (SPSS Inc., Chicago, IL, USA) and statistical significance was set at an alpha level of 0.05.

## Results

### Inter-rater agreement

**Figure 2:**
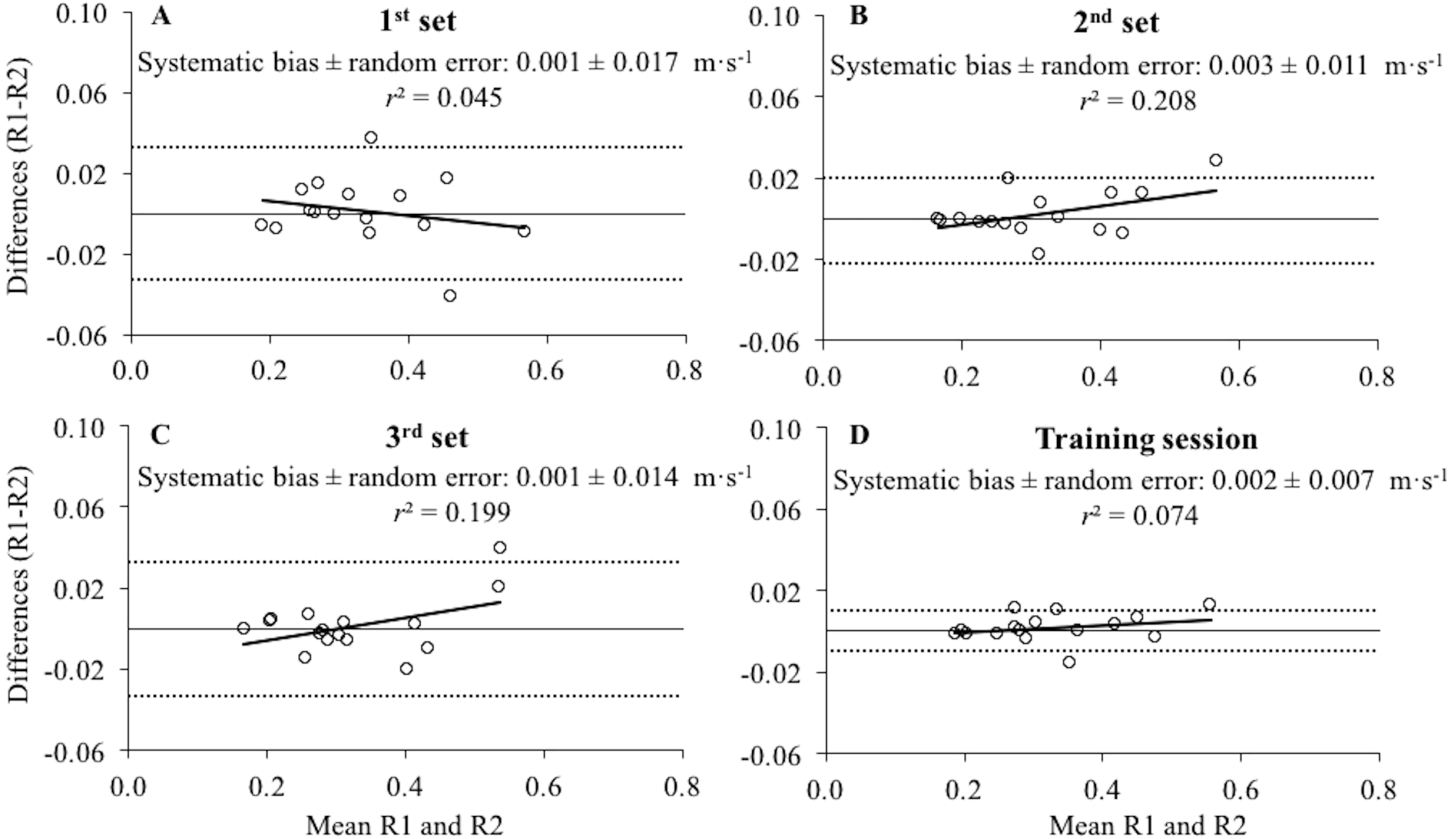
Differences between the raters for the mean velocity values collected during the first, second, third set and the whole training session. Bland-Altman plots showing differences between the raters for the mean velocity values collected during the first set (A), second set (B), third set (C) and the whole training session (average value of the 3 sets; (D). Each plot depicts the averaged difference (straight line) and 95% limits of agreement (dashed lines), along with the regression line. *r*^2^, coefficient of determination.

No significant differences and nearly perfect correlations were observed between raters for the MV values collected under individual sets (*p* ≥ 0.38, *ES* ≤ 0.02, *r* ≥ 0.987) as well as for the whole training session (*p* = 0.38, *ES* = 0.01, *r* = 0.997). Bland-Altman plots also revealed very low systematic bias (≤0.003 m s^−1^) and random errors (≤0.033 m s^−1^), while heteroscedasticity of the errors was observed for the sets 2 (*r*^2^ = 0.208) and 3 (*r*^2^ = 0.199), but not for the set 1 (*r*^2^ = 0.045) or the whole training session (*r*^2^ = 0.074) (Fig. 2).

### iLOAD^®^ app vs. linear encoder

**Figure 3:**
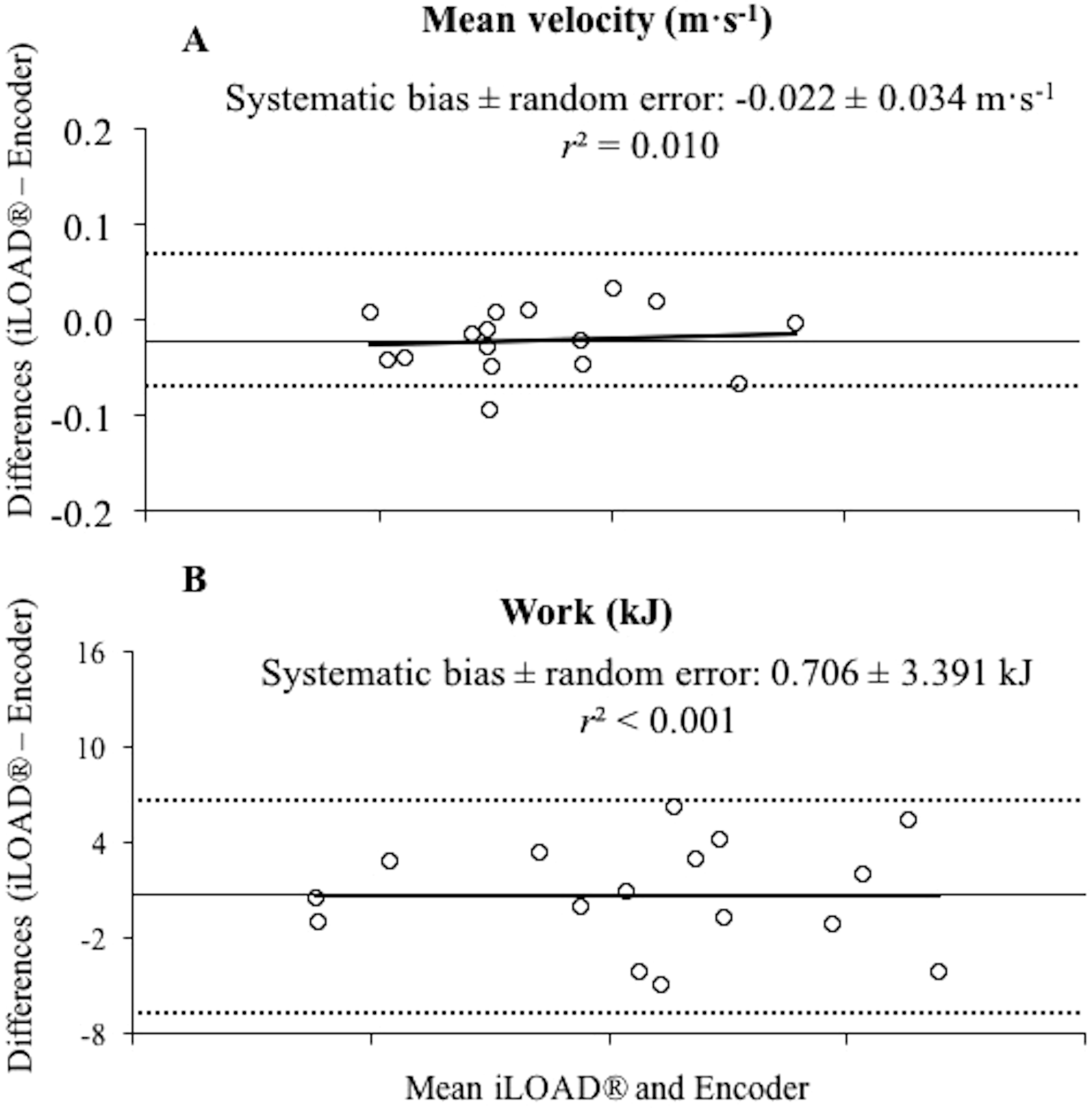
Differences between the *iLOAD^®^* app and the linear encoder for the recordings of mean velocity and total work of the whole training session. Bland-Altman plots showing differences between the *iLOAD^®^* app and the linear encoder for the recordings of mean velocity (A) and total work (B) of the whole training session. Each plot depicts the averaged difference (straight line) and 95% limits of agreement (dashed lines), along with the regression line. *r*^2^, Pearson’s coefficient of determination.

Due to the very high inter-rater agreement reported above, the average value of both raters was considered to explore the concurrent validity of the *iLOAD^®^* app with respect to the linear encoder. Although significant differences between the *iLOAD^®^* app and the linear encoder were observed for MV during the sets 2 and 3 as well as for the whole training session (*p* < 0.05), the magnitude of the differences were trivial-small and the correlations were always nearly perfect between both devices for MV and total work (Table 1). Bland-Altman plots did not reveal heteroscedasticity of the errors between the *iLOAD^®^* app and the linear encoder for MV and total work (*r*^2^ ≤ 0.010) (Fig. 3).

**Table 1:** Comparison of the measurements of mean velocity (MV) and total work (TW) between the *iLOAD^®^* app and a linear encoder.

Variable	Set	iLOAD^®^	Encoder	*p*-value	ES (95% CI)	Pearson’s *r* (95% CI)
MV (m s^−1^)	1st	0.34 ± 0.10	0.35 ± 0.10	0.10	−0.15 (−0.34, 0.03)	0.942 (0.838, 0.980)
	2nd	0.32 ± 0.11	0.34 ± 0.10	0.04	−0.25 (−0.49, −0.01)	0.903 (0.738, 0.966)
	3rd	0.32 ± 0.11	0.35 ± 0.12	0.02	−0.21 (−0.39. −0.04)	0.950 (0.859, 0.982)
	Total	0.32 ± 0.11	0.35 ± 0.10	0.02	−0.21 (−0.39, −0.04)	0.948 (0.854, 0.982)
TW (kJ)	1st	14.9 ± 5.2	14.7 ± 5.2	0.46	0.04 (−0.08, 0.16)	0.975 (0.928, 0.991)
	2nd	14.5 ± 5.6	14.3 ± 5.6	0.49	0.03 (−0.07, 0.14)	0.981 (0.945, 0.993)
	3rd	14.7 ± 5.5	14.4 ± 5.4	0.36	0.05 (−0.07, 0.18)	0.973 (0.922, 0.990)
	Total	44.0 ± 16.1	43.3 ± 16.1	0.42	0.04 (−0.07, 0.16)	0.977 (0.934, 0.992)

**Notes.**

Mean ± standard deviation.

ES effect size

## Discussion

This study was designed to determine the concurrent validity of the *iLOAD^®^* app with respect to a linear encoder (reference method) for monitoring MV and total work during a RT session conducted with the half squat exercise. The experimental data collected in the present study supported our two hypotheses. Namely, the *iLOAD^®^* app showed a very high inter-rater agreement for the recordings of MV, and also a very high validity for the measurements of MV and total work when compared to the data collected with the linear encoder. These results highlight that the *iLOAD^®^* app could be a valuable tool for RT monitoring because it is cheap, easy to use, portable and time-efficient.

Our first hypothesis was confirmed since the inter-rater agreement for the measurement of MV was very high. The inter-rater agreement was comparable to the results reported by Balsalobre-Fernández et al. (2018) for the measurement of MV with *PowerLift^®^* (*p* = 0.549, ICC = 0.941). It should be noted that the main difference between both apps is that *iLOAD^®^* provides the MV of a training set (i.e., from the start of the eccentric phase of the first repetition until the end of the concentric phase of the last repetition) and *PowerLift^®^* reveals the MV of the concentric phase of individual repetitions. Therefore, these apps provide complementary information that could be valuable for prescribing and evaluating the effect of RT programs. Collectively, these results highlight that the outcomes of different smartphone apps specifically designed for monitoring movement velocity should not differ between different evaluators. This result could be expected because smartphone apps (e.g., *iLOAD^®^* and *PowerLift^®^*) are very easy to use. However, it would be important to determine in further studies the potential effect of testers’ experience with the use of smartphone apps on the accuracy of their outcomes.

The linear encoder is frequently considered as the gold-standard for monitoring movement velocity during RT exercises (Balsalobre-Fernández et al., 2018; García-Ramos, Pérez-Castilla & Martín, 2018). Supporting our second hypothesis, the MV recorded with the *iLOAD^®^* app showed a very high level of agreement with respect to the MV collected with the linear encoder. Previous studies have also observed a high validity of *PowerLift^®^* for measuring the MV of individual repetitions during a variety of RT exercises compared to a linear encoder (Balsalobre-Fernández et al., 2017) or a high-speed video camera (Perez-Castilla et al., 2019). Therefore, the results of the present study suggest that smartphone apps are not only useful to determine the MV of individual repetitions (i.e., *PowerLift^®^* app), but also for monitoring the MV of a set of multiple repetitions (*iLOAD^®^* app). However, it should be noted that, in the present study, the MV values collected with the *iLOAD^®^* app were slightly lower compared to the MV values provided by the linear encoder (see Table 1). This result was likely caused because the *iLOAD^®^* app was initiated just after the ’go’ instruction provided by one of the raters. However, the recording of the linear encoder was initiated when a descent of the barbell was detected, and this should have promoted a shorter duration of the training set for the linear encoder because it is expected that the participants started the movement slightly after the *iLOAD^®^* app was initiated. Note that although significant, the magnitude of the differences was trivial-small (ES range = 0.15–0.25). These results suggest that the data collected with the *iLOAD^®^* app should not be used interchangeably with the data provided by a linear encoder when the expected differences are small. Our results reinforce the potential applicability of the *iLOAD^®^* app for monitoring RT based on movement velocity. However, it remains to be elucidated whether the MV of a set may guide coaches and athletes in the same manner as traditional velocity-based training which is based exclusively on the velocity of the concentric phase (González-Badillo, Marques & Sánchez-Medina, 2011; Jovanovic & Flanagan, 2014).

Regarding total work, no significant differences and nearly perfect correlations were found between the *iLOAD^®^* app and the linear encoder in all sets. These results suggest that the total work of a RT session can be accurately quantified with the *iLOAD^®^* app. The only inputs needed by the *iLOAD^®^* app to calculate total work are the vertical displacement of the barbell during a single repetition, the user body mass, the load lifted, and the number of repetitions performed. To our knowledge, *iLOAD^®^* is the first smartphone app that has been designed to quantify total work during RT sessions. The recording of total work is important because it is considered as one of the most objective measures to quantify the total volume during RT, and it is also one of the most appropriate methods for equating training volume in different RT exercises (Cormie, McCaulley & McBride, 2007; McCaulley et al., 2007; McBride et al., 2009). Note that two athletes with different heights but similar body mass would complete different amount of work for the same load (kg) during the half squat exercise because the distance completed in each repetition directly influences the total work performed. Therefore, the *iLOAD^®^* app allows practitioners to obtain real-time accurate measures of the total work performed during a RT session. This augmented feedback may help to improve both physical performance (Weakley et al., in press) and psychological traits (Wilson et al., 2017) in athletes whilst training.

The use of the *iLOAD^®^* app is not without limitations. The main issue related to the *iLOAD^®^* app is that the MV encompasses the whole training set and not only the MV of the concentric phase of individual repetitions. Note that velocity-based RT guidelines has been proposed considering only the MV of the concentric phase of individual repetitions (González-Badillo, Marques & Sánchez-Medina, 2011; Jovanovic & Flanagan, 2014). Therefore, future studies should elucidate whether the MV of the training set provided by the *iLOAD^®^* app could also provide valuable information to prescribe and monitor RT programs. Another important issue is that we only examined the validity of the *iLOAD^®^* app for sets consisting of approximately 10 repetitions and, consequently, future studies should clarify whether the *iLOAD^®^* app can also provide accurate data when performing a lower number of repetitions. Finally, for testing purposes, it would also be important to determine the reliability of *iLOAD^®^* app for the measurement of MV during sets consisting of different number of repetitions in a variety of RT exercises.

## Conclusions

The main finding of the present study is that the *iLOAD^®^* app showed a high validity for monitoring the MV and total work in a set of multiple repetitions during the half squat exercise. Therefore, the *iLOAD^®^* app can be considered as a cheap, easy to use, portable and time-efficient tool for RT monitoring. Future studies should explore the validity of the *iLOAD^®^* app for RT monitoring with other RT exercises.

##  Supplemental Information

10.7717/peerj.7372/supp-1 Supplemental Information 1 Suplemental file Data used for statistical analysis

10.7717/peerj.7372/supp-2 Supplemental Information 2 Application code Zip file with code.

## Additional Information and Declarations

### Data Availability

The following information was supplied regarding data availability: Raw data is available in the Supplemental Files.
