# Supplementary figures and images for: Validity of the iLOAD® app for resistance training monitoring
Source: PeerJ. 2019 Aug 7;7:e7372. doi: 10.7717/peerj.7372 (PMC6689219; doi:10.7717/peerj.7372)

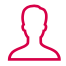

Supplement: Supplemental Information 2 — Zip file with code. [file peerj-07-7372-s002.zip › iLoad/iLoadProject/Assets.xcassets/contact.imageset/User-66.png]

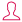

Supplement: Supplemental Information 2 — Zip file with code. [file peerj-07-7372-s002.zip › iLoad/iLoadProject/Assets.xcassets/contact.imageset/User-22.png]

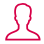

Supplement: Supplemental Information 2 — Zip file with code. [file peerj-07-7372-s002.zip › iLoad/iLoadProject/Assets.xcassets/contact.imageset/User-44.png]

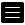

Supplement: Supplemental Information 2 — Zip file with code. [file peerj-07-7372-s002.zip › iLoad/iLoadProject/Assets.xcassets/ActivitiesFilled.imageset/Activity Feed Filled-25.png]

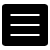

Supplement: Supplemental Information 2 — Zip file with code. [file peerj-07-7372-s002.zip › iLoad/iLoadProject/Assets.xcassets/ActivitiesFilled.imageset/Activity Feed Filled-50.png]

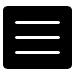

Supplement: Supplemental Information 2 — Zip file with code. [file peerj-07-7372-s002.zip › iLoad/iLoadProject/Assets.xcassets/ActivitiesFilled.imageset/Activity Feed Filled-75.png]

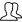

Supplement: Supplemental Information 2 — Zip file with code. [file peerj-07-7372-s002.zip › iLoad/iLoadProject/Assets.xcassets/contacts.imageset/User Group Man Man-22.png]

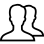

Supplement: Supplemental Information 2 — Zip file with code. [file peerj-07-7372-s002.zip › iLoad/iLoadProject/Assets.xcassets/contacts.imageset/User Group Man Man-44.png]

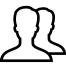

Supplement: Supplemental Information 2 — Zip file with code. [file peerj-07-7372-s002.zip › iLoad/iLoadProject/Assets.xcassets/contacts.imageset/User Group Man Man-66.png]

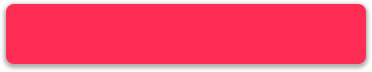

Supplement: Supplemental Information 2 — Zip file with code. [file peerj-07-7372-s002.zip › iLoad/iLoadProject/Assets.xcassets/bot_finalizar_exercicio.imageset/bot_finalizar_exercicio.png]

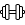

Supplement: Supplemental Information 2 — Zip file with code. [file peerj-07-7372-s002.zip › iLoad/iLoadProject/Assets.xcassets/Exercise.imageset/Dumbbell-25.png]

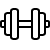

Supplement: Supplemental Information 2 — Zip file with code. [file peerj-07-7372-s002.zip › iLoad/iLoadProject/Assets.xcassets/Exercise.imageset/Dumbbell-50.png]

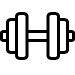

Supplement: Supplemental Information 2 — Zip file with code. [file peerj-07-7372-s002.zip › iLoad/iLoadProject/Assets.xcassets/Exercise.imageset/Dumbbell-76.png]

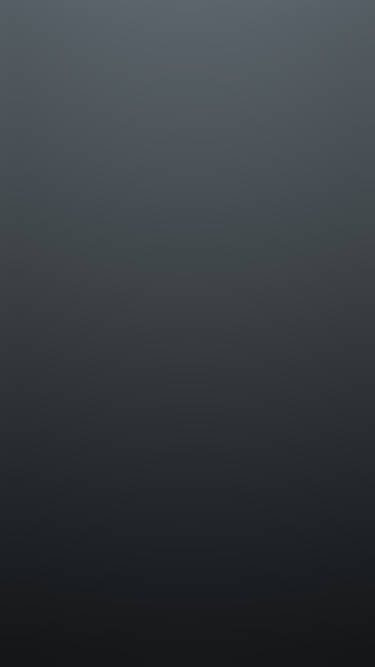

Supplement: Supplemental Information 2 — Zip file with code. [file peerj-07-7372-s002.zip › iLoad/iLoadProject/Assets.xcassets/fundo_app.imageset/Rectangle 2.png]

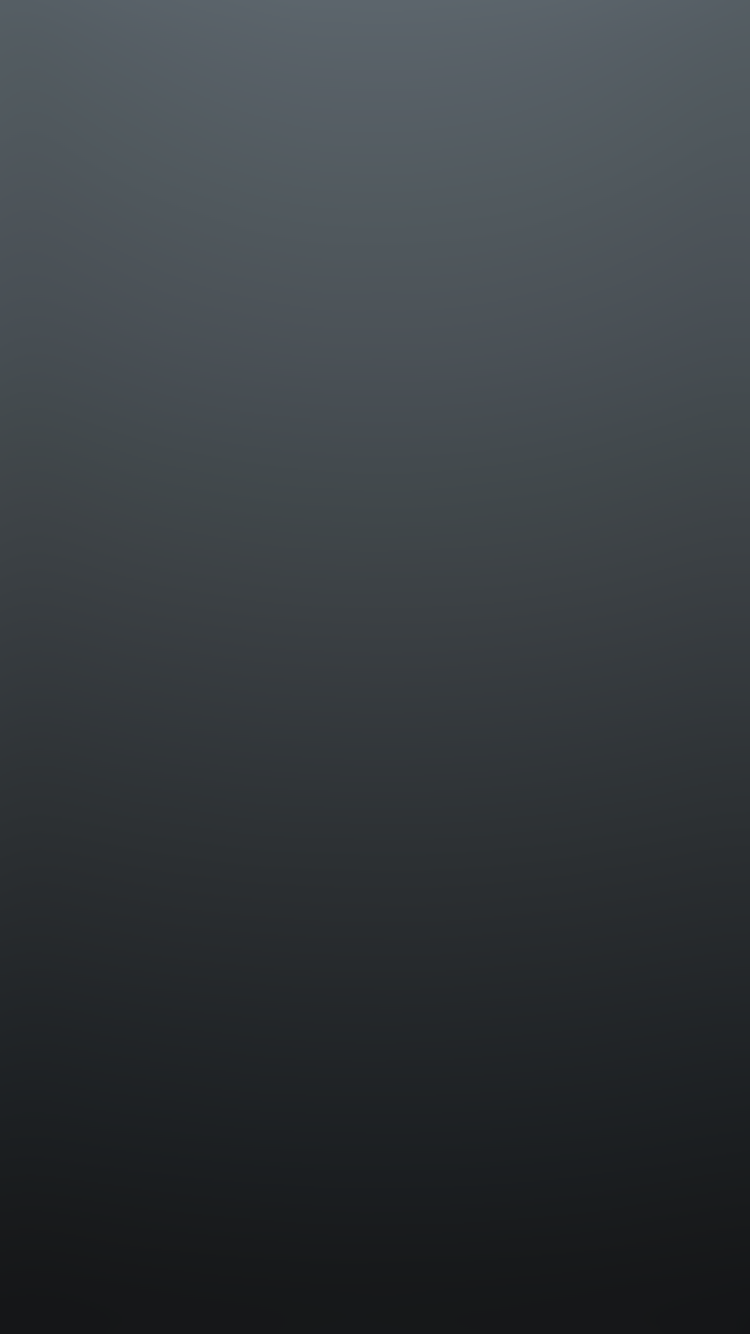

Supplement: Supplemental Information 2 — Zip file with code. [file peerj-07-7372-s002.zip › iLoad/iLoadProject/Assets.xcassets/fundo_app.imageset/Rectangle 2@2x.png]

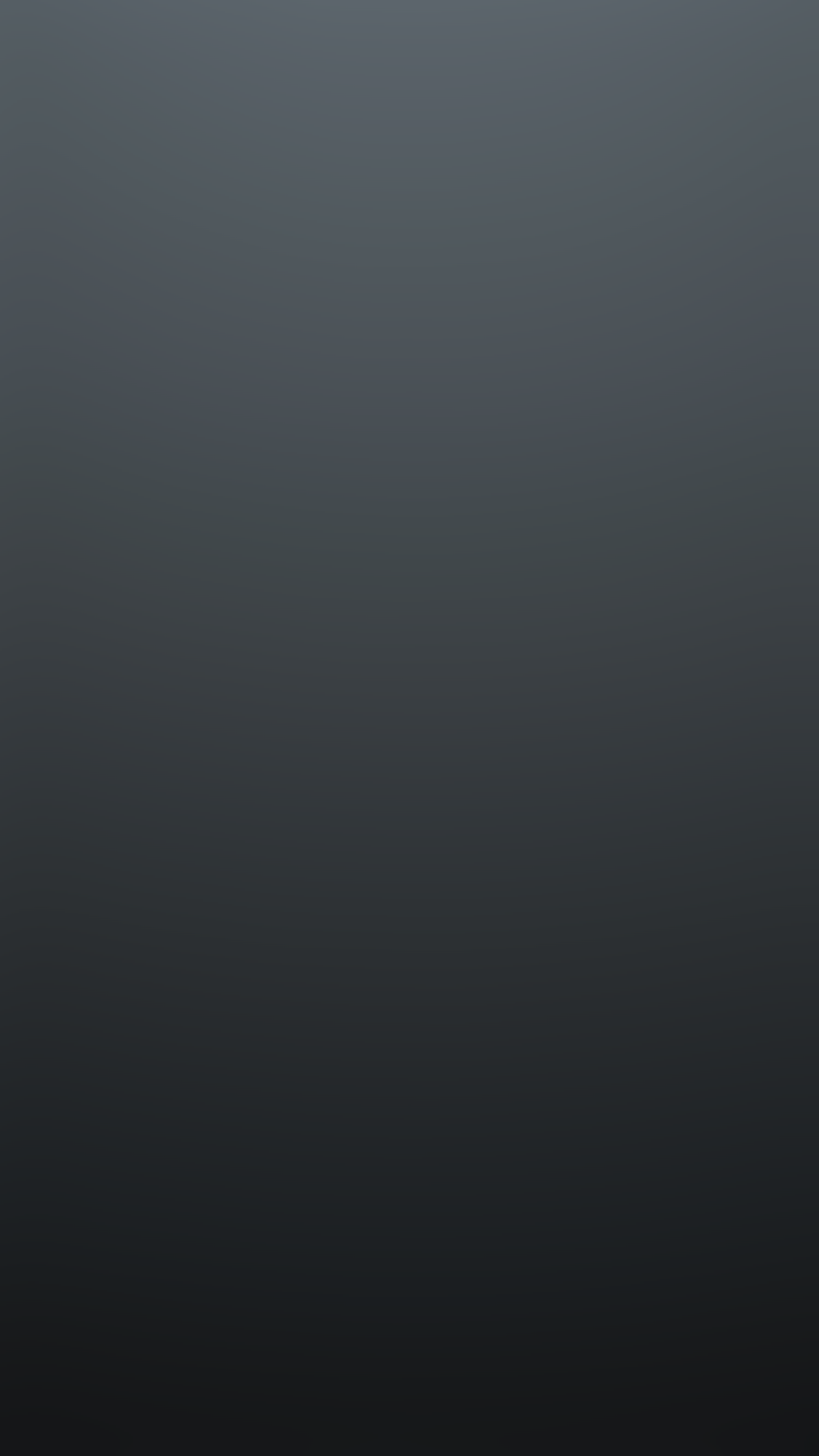

Supplement: Supplemental Information 2 — Zip file with code. [file peerj-07-7372-s002.zip › iLoad/iLoadProject/Assets.xcassets/fundo_app.imageset/Rectangle 2@3x.png]

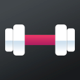

Supplement: Supplemental Information 2 — Zip file with code. [file peerj-07-7372-s002.zip › iLoad/iLoadProject/Assets.xcassets/AppIcon.appiconset/iloadicone-Small-40@2x-1.png]

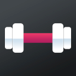

Supplement: Supplemental Information 2 — Zip file with code. [file peerj-07-7372-s002.zip › iLoad/iLoadProject/Assets.xcassets/AppIcon.appiconset/iloadicone-76.png]

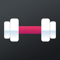

Supplement: Supplemental Information 2 — Zip file with code. [file peerj-07-7372-s002.zip › iLoad/iLoadProject/Assets.xcassets/AppIcon.appiconset/iloadicone-60.png]

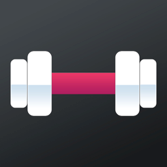

Supplement: Supplemental Information 2 — Zip file with code. [file peerj-07-7372-s002.zip › iLoad/iLoadProject/Assets.xcassets/AppIcon.appiconset/iloadicone-83.5@2x.png]

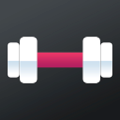

Supplement: Supplemental Information 2 — Zip file with code. [file peerj-07-7372-s002.zip › iLoad/iLoadProject/Assets.xcassets/AppIcon.appiconset/iloadicone-Small-40@3x.png]

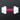

Supplement: Supplemental Information 2 — Zip file with code. [file peerj-07-7372-s002.zip › iLoad/iLoadProject/Assets.xcassets/AppIcon.appiconset/iloadicone-Small-20.png]

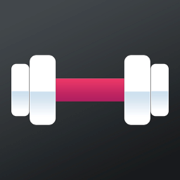

Supplement: Supplemental Information 2 — Zip file with code. [file peerj-07-7372-s002.zip › iLoad/iLoadProject/Assets.xcassets/AppIcon.appiconset/iloadicone-60@3x.png]

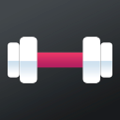

Supplement: Supplemental Information 2 — Zip file with code. [file peerj-07-7372-s002.zip › iLoad/iLoadProject/Assets.xcassets/AppIcon.appiconset/iloadicone-60@2x.png]

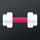

Supplement: Supplemental Information 2 — Zip file with code. [file peerj-07-7372-s002.zip › iLoad/iLoadProject/Assets.xcassets/AppIcon.appiconset/iloadicone-Small-42.png]

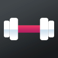

Supplement: Supplemental Information 2 — Zip file with code. [file peerj-07-7372-s002.zip › iLoad/iLoadProject/Assets.xcassets/AppIcon.appiconset/iloadicone-Small@2x.png]

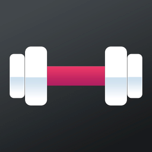

Supplement: Supplemental Information 2 — Zip file with code. [file peerj-07-7372-s002.zip › iLoad/iLoadProject/Assets.xcassets/AppIcon.appiconset/iloadicone-76@2x.png]

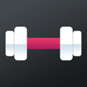

Supplement: Supplemental Information 2 — Zip file with code. [file peerj-07-7372-s002.zip › iLoad/iLoadProject/Assets.xcassets/AppIcon.appiconset/iloadicone-Small@3x.png]

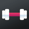

Supplement: Supplemental Information 2 — Zip file with code. [file peerj-07-7372-s002.zip › iLoad/iLoadProject/Assets.xcassets/AppIcon.appiconset/iloadicone-Small.png]

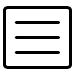

Supplement: Supplemental Information 2 — Zip file with code. [file peerj-07-7372-s002.zip › iLoad/iLoadProject/Assets.xcassets/Activities.imageset/Activity Feed-75.png]

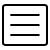

Supplement: Supplemental Information 2 — Zip file with code. [file peerj-07-7372-s002.zip › iLoad/iLoadProject/Assets.xcassets/Activities.imageset/Activity Feed-50.png]

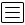

Supplement: Supplemental Information 2 — Zip file with code. [file peerj-07-7372-s002.zip › iLoad/iLoadProject/Assets.xcassets/Activities.imageset/Activity Feed-25.png]

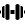

Supplement: Supplemental Information 2 — Zip file with code. [file peerj-07-7372-s002.zip › iLoad/iLoadProject/Assets.xcassets/ExerciseFilled.imageset/Dumbbell Filled-25.png]

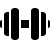

Supplement: Supplemental Information 2 — Zip file with code. [file peerj-07-7372-s002.zip › iLoad/iLoadProject/Assets.xcassets/ExerciseFilled.imageset/Dumbbell Filled-50.png]

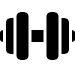

Supplement: Supplemental Information 2 — Zip file with code. [file peerj-07-7372-s002.zip › iLoad/iLoadProject/Assets.xcassets/ExerciseFilled.imageset/Dumbbell Filled-75.png]

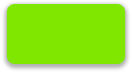

Supplement: Supplemental Information 2 — Zip file with code. [file peerj-07-7372-s002.zip › iLoad/iLoadProject/Assets.xcassets/bot_comecar.imageset/bot_comecar.png]

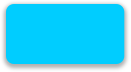

Supplement: Supplemental Information 2 — Zip file with code. [file peerj-07-7372-s002.zip › iLoad/iLoadProject/Assets.xcassets/bot_repetir.imageset/bot_repetir.png]

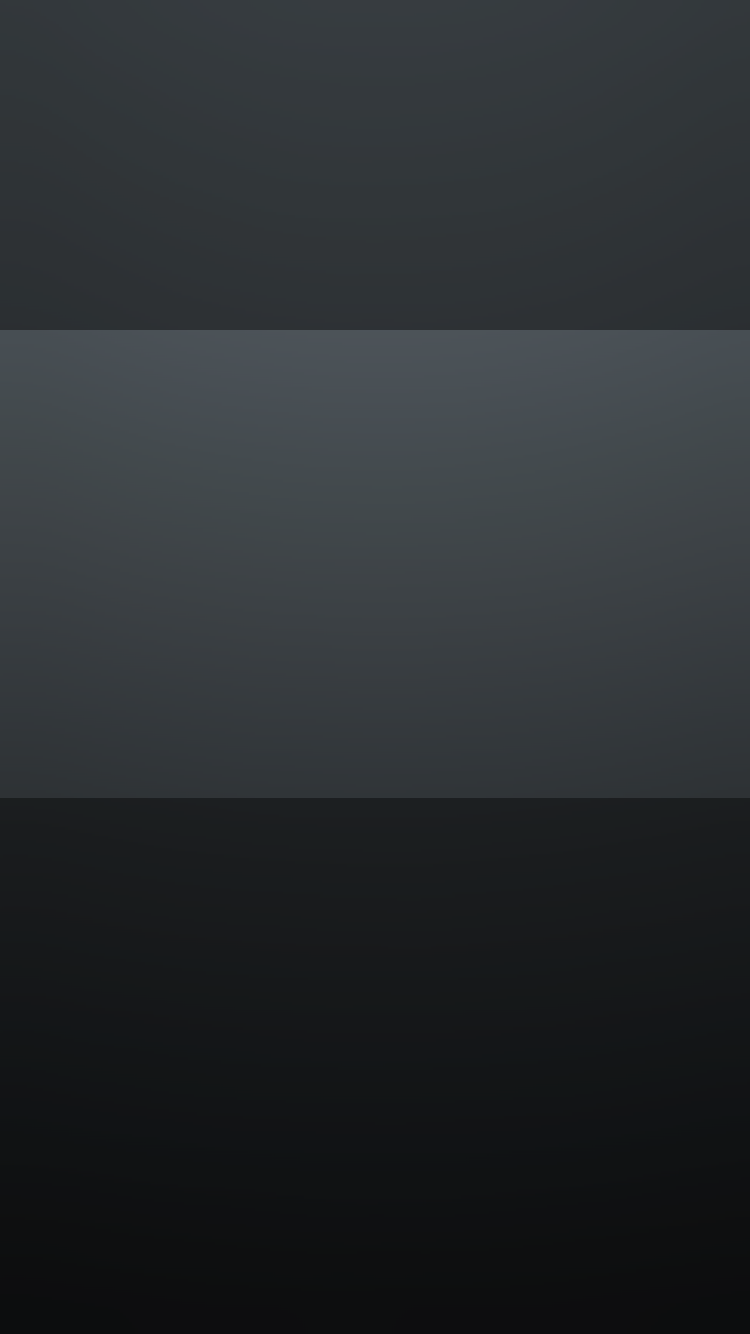

Supplement: Supplemental Information 2 — Zip file with code. [file peerj-07-7372-s002.zip › iLoad/iLoadProject/Assets.xcassets/fundo_app1.imageset/fundo_app.png]
